# RNA isolation for transcriptomics of human and mouse small skin biopsies

**DOI:** 10.1186/1756-0500-4-438

**Published:** 2011-10-24

**Authors:** Oskar Bruning, Wendy Rodenburg, Teodora Radonic, Aeilko H Zwinderman, Annemieke de Vries, Timo M Breit, Mark de Jong

**Affiliations:** 1 MicroArray Department & Integrative Bioinformatics Unit (MAD-IBU), Swammerdam Institute for Life Sciences (SILS); Faculty of Science (FNWI), University of Amsterdam (UvA), Science Park 904, 1098 XH Amsterdam, The Netherlands; 2 Laboratory for Health Protection Research (GBO), National Institute of Public Health and the Environment (RIVM), Antonie van Leeuwenhoeklaan 9, 3721 MA Bilthoven, The Netherlands; 3 Department of Clinical Epidemiology, Biostatistics and Bioinformatics, Academic Medical Center (AMC) Amsterdam, Meibergdreef 9, 1105 AZ, Amsterdam, The Netherlands

## Abstract

**Background:**

Isolation of RNA from skin biopsies presents a challenge, due to the tough nature of skin tissue and a high presence of RNases. As we lacked the dedicated equipment, i.e. homogenizer or bead-beater, needed for the available RNA from skin isolation methods, we adapted and tested our zebrafish single-embryo RNA-isolation protocol for RNA isolation from skin punch biopsies.

**Findings:**

We tested our new RNA-isolation protocol in two experiments: a large-scale study with 97 human skin samples, and a small study with 16 mouse skin samples. Human skin was sampled with 4.0 mm biopsy punches and for the mouse skin different punch diameter sizes were tested; 1.0, 1.5, 2.0, and 2.5 mm. The average RNA yield in human samples was 1.5 μg with an average RNA quality RIN value of 8.1. For the mouse biopsies, the average RNA yield was 2.4 μg with an average RIN value of 7.5. For 96% of the human biopsies and 100% of the mouse biopsies we obtained enough high-quality RNA. The RNA samples were successfully tested in a transcriptomics analysis using the Affymetrix and Roche NimbleGen platforms.

**Conclusions:**

Using our new RNA-isolation protocol, we were able to consistently isolate high-quality RNA, which is apt for further transcriptomics analysis. Furthermore, this method is already useable on biopsy material obtained with a punch diameter as small as 1.5 mm.

## Background

The use of skin biopsies is an important method for studying the *in vivo *effects of harmful agents or radiation on skin tissue as well as skin diseases. However, for -omics experiments such as transcriptomics, it is often difficult to obtain sufficient RNA from biopsies. This is primarily caused by the tough nature of skin material, which makes it hard to homogenize a skin sample, in addition to the high number of RNases in skin tissue that quickly degrade the biopsy RNA [1-5]. Because of this, several biopsies often need to be pooled, which can lead to dilution of effects by biological variation plus various other unwanted effects [6-8]. More or larger biopsies will increase the discomfort for human test subjects or the required number of test animals. More efficient ways of isolating RNA from this limited sample material holds the promise of decreasing the biopsy-related human discomfort and numbers of test animals.

The limited amount of biopsy material from an experiment of 97 human skin biopsies, i.e. half of a 4.0 mm diameter biopsy, initiated the development of an optimal skin RNA-isolation protocol. At the same time, we investigated whether such a protocol would also allow for an experimental set-up with multiple skin biopsies per mouse, as this would imply the use of small biopsy punches (2.5 mm - 1.0 mm). Because, neither bead-beater nor homogenizer, which are commonly used for this type of RNA extraction [1,2], are available in our setting, we decided to adapt our previously described robust and highly-efficient protocol for RNA extraction from single zebrafish embryos [9]. This protocol is based on sample homogenization in liquid nitrogen, RNA extraction by phenol and column purification. By adapting the zebrafish protocol we anticipated to be able to isolate, in an affordable way with standard equipment available in most molecular biology laboratories, enough RNA material from these biopsies with the required quality for use in -omics experiments.

## Results

After testing of several adaptations on our zebrafish single-embryo protocol, it showed optimal performance by the addition of a ten minutes shake step (Additional file 1, step11) [9].

To evaluate our skin-biopsy RNA-isolation protocol, we defined five key criteria: 1) Weight of the biopsy; 2) RNA yield; 3) RNA Integrity Number (RIN [10]) value for RNA quality; 4) aRNA yield after *in-vitro *amplification (IVT); and 5) average probe-signal intensity level after microarray analysis. We performed a human and a mouse study to assess the performance of our new RNA-isolation protocol.

**Figure 1:**
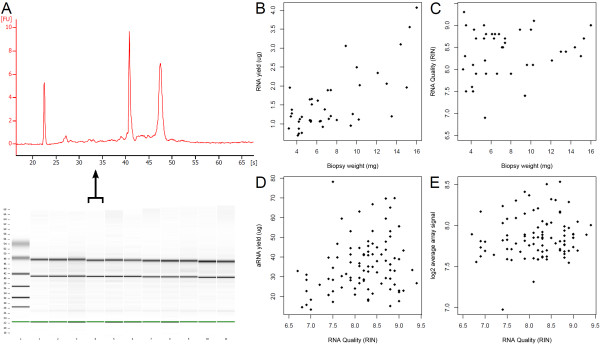
**Scatterplots Human biopsies**. (A) Representative bioanalyzer results showing RNA quality from RNA isolated from 11 human biopsies. (B) Scatterplot showing the linear relationship between biopsy weight and RNA yield for 44 human biopsies. (C) Scatterplot of biopsy weight versus RNA quality for 44 human biopsies showing no clear relationship, although heavier biopsies appear to have less spread in RIN value than lighter samples. (D) Scatterplot of RNA quality versus the aRNA yield for 97 human biopsies showing no clear-cut relation. (E) Scatterplot of RNA quality versus average probe signal for 97 human biopsies showing no clear-cut relation. For the plots in B, C, D and E extreme outliers were removed that bias the scale too much. Scatterplots containing these outliers are shown in Additional file 3

For the experiment with 97 human patients, skin biopsies were taken with a 4.0 mm biopsy punch. Tissue material from these biopsies was divided and one half was used for RNA extraction, while the other half was used for pathologic examination. The results of the five protocol-evaluation criteria are summarized in Table 1. Applying the Affymetrix protocol thresholds (Table 1), 100% of the 97 biopsies yielded sufficient RNA and only 4 biopsies had a RIN value below the 6.5 threshold. Thus 96% of the human skin biopsies displayed high enough quality for further processing. An example of the RNA quality is shown in Figure 1A.

**Table 1:** Summary of evaluation criteria for the RNA-isolation protocol

Experiment	#	Biopsy weight*		RNA yield		RIN value		aRNA yield**^$^		Array signal**^$¥^	
**threshold**		**-**		**0.1 μg Affymetrix** **0.2 μg NimbleGen**		**6.5 for both platforms**		**10.0 μg Affymetrix** **2.0 μg NimbleGen**		**-**	

		**avg**.	**min**.	**avg**.	**min**.	**avg**.	**min**.	**avg**.	**min**.	**avg**.	**min**.

Human	97	8.2 mg	3.2 mg	1.5 μg	0.2 μg	8.1	3.3	35.6 μg	10.0 μg	7.8	5.6

Mouse	16	ND	ND	2.4 μg	0.6 μg	7.5	6.5	4.2 μg	2.9 μg	11.0	10.5

ND: not determined

* 44 half human biopsies were weighted.

** 12 mouse biopsies were analyzed by microarray technology

^$ ^Platform dependent results and thresholds.

^¥ ^Array averaged log2 probe intensity

To investigate the relationship between the protocol-evaluation criteria we plotted the results of the individual biopsies against each other (Figure 1 and Additional file 2, 3). As expected, there appears to be a linear relationship between the weight of the starting biopsy material and the RNA yield (Figure 1B). The RNA quality is consistently high, however, with heavier biopsies, the spread in RNA quality is smaller (Figure 1C). As equal amounts of input RNA or aRNA were used in the IVT reaction and microarray protocol, respectively, the relation between the biopsy weight and aRNA yield or average probe signal gets lost. There seems to be no obvious relation between RNA quality and aRNA yield or average probe signal (Figure 1D and 1E).

After successful extraction of RNA from small human skin biopsies, we tested the performance of our new protocol in a limited experiment with mouse skin biopsies from test animals (un-)treated with UV light radiation (Additional file 4). Here we determined the minimal diameter size of biopsy punch that can be used to consistently obtain sufficient RNA from a skin biopsy for transcriptome analysis. This experiment consisted of 16 skin biopsies from four, approximately four months old, mice, obtained by biopsy punches of different sizes within one minute after they were euthanized. The examined punch diameters were: 1.0, 1.5, 2.0, and 2.5 mm. As mouse skin is less firm than human skin, we had to fold the skin while punching. For the 1.0 and 1.5 mm diameter punches the biopsies thus resulted in two small holes (Additional file 5). For the 2.0 and 2.5 mm diameter punches a half moon shape biopsy on folded skin was made, resulting in one round hole (Additional file 5). Since the obtained mouse biopsies were too small for a proper weight measurement, the punch diameter size was used as an indicator for the amount of material.

**Figure 2:**
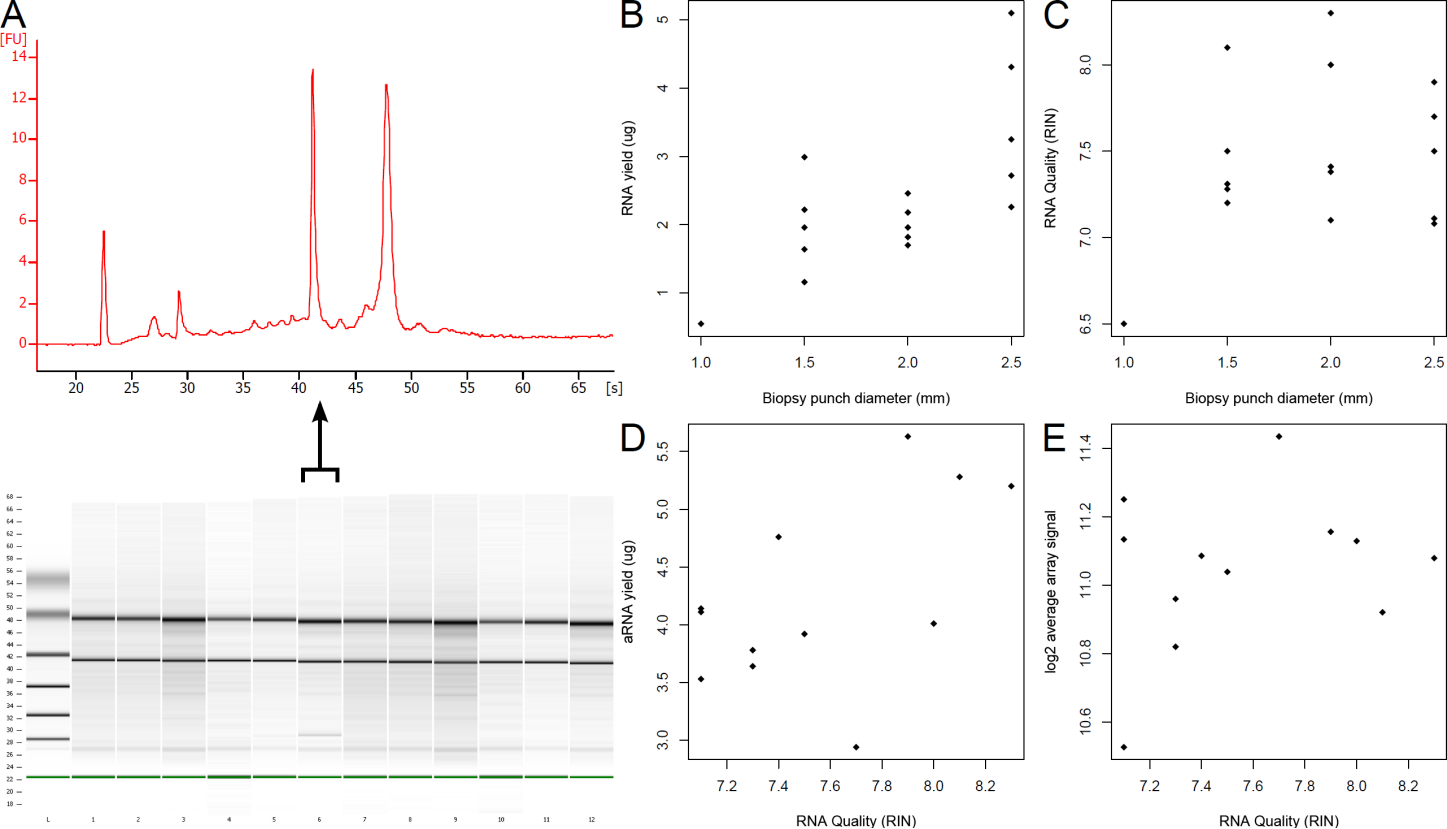
**Scatterplots Mouse biopsies**. (A) Representative bioanalyzer results showing RNA quality from RNA isolated from 12 mouse biopsies. (B) Scatterplot showing the linear relationship between biopsy weight and RNA yield for 16 mouse biopsies. (C) Scatterplot of biopsy diameter versus RNA quality for 16 mouse biopsies showing no clear relationship. (D) Scatterplot of RNA quality versus the aRNA yield for 16 mouse biopsies showing somewhat linear relation. (E) Scatterplot of RNA quality versus the average probe signal for 16 mouse biopsies showing no clear-cut relation.

The results of this experiment are summarized in Table 1. RNA from all these biopsies had a RIN value of at least the 6.5 threshold, meaning that 100% of the RNA samples could be used for further processing. An example of the RNA quality is shown in Figure 2A. The differences in scale, present between the human and mouse studies in aRNA yield and average probe signals, are caused by differences in the microarray platforms employed in the two experiments (Table 1).

To investigate our protocol-evaluation criteria, we plotted the results for the individual biopsies (Figure 2 and Additional file 4). The relationship between the biopsy punch diameter and RNA yield is linear (Figure 2B). The RNA quality is constantly high, with no clear differences between diameter sizes, except for the 1.0 mm biopsy (Figure 1C). The resulting biopsy material from this punch diameter size was too small for proper and practical handling and this settled the lower punch diameter limit to 1.5 mm. Therefore, the 1.0 mm biopsy was left out of the microarray analysis.

**Figure 3:**
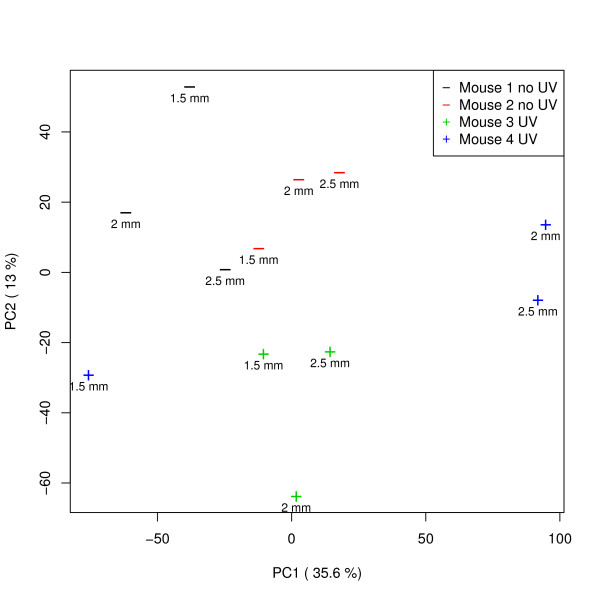
**Principal components analysis of mouse microarray results**. Principal components analysis (PCA) of the non-normalized log2 ratios of (biopsy/reference) showing a clear separation of the UV treated biopsies (+) from the untreated (-). The punch size seems to have no effect on these first two principal component axis. An effect from the animal that was used, is apparent.

There seems to be a fairly linear relationship between RNA quality and aRNA yield for these biopsies (Figure 2D). However, the average probe signal seems to have no clear relation with the RNA quality (Figure 2E). For an impression of the performance of our RNA-isolation protocol in transcriptomics, we analyzed the microarray results by principal components analysis (PCA) (Figure 3). The PCA result shows a clear separation of UV-treated versus non-treated samples and an effect for test-animal. Only the biopsy 1.5 mm sample from mouse number 4 is relatively far away from the other samples from this mouse on the PCA1 axis. This is most likely due to the fact that the RNA yield is quite low compared to all other samples (Additional file 4), which appears to have some effect on the test-animal effect. This indicates that the differences in biopsy punch diameter overall have a smaller effect on gene expression than the variable "test-animal" or experimental treatment with UV light.

## Conclusions

Our RNA-isolation protocol for human and mouse small skin biopsies presented here robustly produces sufficient good quality RNA for transcriptomics experimentation. Although our protocol results in similar RNA yields as compared to other RNA-isolation protocols [1,2] (Additional file 6), the absence for dedicated laboratory equipment renders the advantage that it is easy to implement.

Furthermore, sufficient RNA can reproducibly be obtained by using a very small biopsy punch diameter of just 1.5 mm. For human skin studies this may imply that skin tissue can be obtained with significantly less discomfort for the test individuals. With respect to mouse experimentation, our results indicate that animals do not need to be sacrificed each time *in vivo *skin material is needed. As such, skin biopsies up to possibly six biopsies per animal could also be used in longitudinal studies, requiring significantly less animals, while increasing the statistical power by reducing the biological variation.

## Methods

### Biological material from humans

Skin biopsies with a 4.0 mm diameter punch were taken from the upper thigh or upper arm of 97 patients, before and 4 weeks after therapy with a drug to treat high blood pressure, after local anesthesia with ethyl chloride spray. Biopsies were immediately snap frozen in liquid nitrogen and stored at -80°C until further processing.

The protocol has been approved by the Medical Ethical Committee of the Academic Medical Center in Amsterdam. The feasibility approvals have been obtained from all the participating centers. This trial is registered in the Netherlands Trial Register under number NTR1423. Enrollment began in March 2008 and in October 2009 230 patients have been enrolled.

### Biological material from mice

Four 3 to 4 months old SKH1 hairless mice were used in this experiment: two were untreated controls and two were irradiated with 300 J/m^2 ^UV B light. 24 hours after exposure, both treated and untreated mice were euthanized by cervical dislocation. This was followed directly by biopsy sampling from the back to prevent RNA decay. For one mouse, biopsies were also taken from the abdomen. Biopsies were immediately snap frozen in liquid nitrogen and stored at -80°C until further processing.

Biopsies using a 1.0 mm diameter punch were unpractical as part of the material remained inside the skin upon punching. The 1.0 and 1.5 mm biopsies were taken by punching through the folded skin resulting in two holes and the 2.0 and 2.5 mm ones by punching a half moon shape on folded skin resulting in one round hole.

The study was agreed upon by the institute's Experimental Animal Ethical Committee and carried out in accordance with national legislation.

### RNA isolation

Per RNA isolation, one 1.5 ml tube was filled beforehand with 75-100 mg phase-lock gel heavy (5-Prime) and pelleted for 30 s at 12,000 × g. Single biopsies were pulverized to a fine powder with a standard liquid nitrogen pre-chilled mortar and pestle. This powder was transferred to a 1.5 ml tube with 300 μl Qiazol (Qiagen). The homogenate was vortexed vigorously for 15 s and subsequently shaken for 10 min on a REAX 2000 (Heidolph). After a quick spin-down 60 μl chloroform was added to the homogenate, vortexed for 15 s and kept at room temperature for 3 min. The partly separated mixture was transferred as a whole to a pre-prepared phase-lock gel heavy containing tube and centrifuged for 15 min at 12,000 × g. The aqueous phase was transferred to a new 1.5 ml tube. RNA was purified by column precipitation according to the RNeasy MinElute Cleanup Handbook (version 2007) - Appendix D: RNA Cleanup after Lysis and Homogenization with Qiazol Lysis Reagent (Qiagen). At the end of the procedure, the RNA was eluted in 14 μl nuclease-free water. A PDF file with the complete protocol is available in a convenient format in the Additional file 1.

### Microarrays with Human Affymetrix platform

Gene expression was analyzed with Human Exon 1.0 ST Arrays in biopsies from all 97 patients. Sense-strand cDNA was generated from total RNA using Ambion WT Expression Kit (Applied Biosystems) conform the manufacturer's instructions. Further steps were performed using the manufacturer's protocols for the GeneChip platform (Affymetrix). Those included purification of double-stranded cDNA, synthesis of aRNA by in vitro transcription, recovery and quantification of biotin-labeled aRNA, fragmentation of this aRNA and subsequent hybridization to the microarray, post-hybridization washings and detection of the hybridized aRNA using a streptavidin-coupled fluorescent dye. Hybridized Affymetrix GeneChips were scanned using a GeneChip Scanner 3000-7G (Affymetrix). Image generation and feature extraction were performed using Affymetrix GCOS Software v1.4.0.036.

### Microarrays with Mouse Roche NimbleGen platform

Gene expression of mouse samples was analyzed with a 12 × 135 k *Mus musculus *microarray (Catalog no. 05543797001, Design 090901 MM9 EXP HX12) containing 44,170 genes with 3 probes per target gene. Per RNA sample, 200 ng total RNA was amplified according to the Agilent LRILAK kit manual (Agilent technologies). Amino-allyl modified nucleotides were incorporated during the aRNA synthesis (2.5 mM rGAU (GE Healthcare), 0.75 mM rCTP (GE Healthcare), 0.75 mM AA-rCTP (TriLink Biotechnologies). Synthesized aRNA was purified with the E.Z.N.A. MicroElute RNA Clean Up Kit (Omega Bio-Tek). Test aRNA samples were labeled with Cy3 and a Reference sample (made by pooling equimolar amounts of RNA from Test samples) was labeled with Cy5. 5 μg of aRNA was dried down and dissolved in 50 mM carbonate buffer pH 8.5. Individual vials of Cy3/Cy5 from the mono-reactive dye packs (GE Healthcare) were dissolved in 200 μl DMSO. To each sample, 10 μl of the appropriate CyDye dissolved in DMSO was added and the mixture was incubated for 1 h. Reactions were quenched with the addition of 5 μl 4 M hydroxylamine (Sigma-Aldrich). The labeled aRNA was purified with the E.Z.N.A. MicroElute RNA Clean Up Kit. The yields of aRNA and CyDye incorporation were measured on the NanoDrop ND-1000.

Each hybridization mixture was made up from 1.1 μg Test (Cy3) and 1.1 μg Reference (Cy5) sample. Samples were dried and 1.98 μl of water was added. The hybridization cocktail was made according to the manufacturer's instructions (Roche NimbleGen Arrays User's Guide - Gene Expression Arrays Version 5.0, Roche NimbleGen). 5.22 μl from this mix was added to each sample. The samples were incubated for 5 min at 65°C and 5 min at 42°C prior to loading. Hybridization samples were loaded onto the microarrays, and hybridized for 18 hours at 42°C with the Roche NimbleGen Hybridization System 4. Afterwards, the slides were washed according to the Roche NimbleGen Arrays User's Guide - Gene Expression Arrays Version 5.0 and scanned in an ozone-free room with a DNA microarray scanner G2565CA (Agilent Technologies). Feature extraction was performed with NimbleScan v2.5 (Roche NimbleGen). The array data have been deposited in NCBI's Gene Expression Omnibus and is accessible through GEO Series accession number GSE28463 http://www.ncbi.nlm.nih.gov/geo/.

### Data analysis

To generate the average log2 probe signal for the Affymetrix GeneChips, raw probe intensities without control probes were used. For the Nimblegen microarrays, raw sample channel data without control probes was used. Data handling, scatterplot generation and PCA analysis were performed using R-2.11.1 http://www.R-project.org and Bioconductor http://www.bioconductor.org/ software.

## Competing interests

The authors declare that they have no competing interests.

## Authors' contributions

OB performed the data analysis and drafted the manuscript. WR and AdV provided the mouse biopsies. TR and AHZ provided the human biopsies. TMB helped to draft the manuscript. MdJ conceived of the study, and participated in its design and coordination and helped to draft the manuscript. All authors read and approved the final manuscript.

## Supplementary Material

Additional file 1 **RNA isolation protocol**. Step-by-step protocol describing the isolation of RNA from skin biopsies. Click here for file

Additional file 2 **Human biopsies results**. Table showing the quality measure results for individual RNA samples isolated from human skin biopsies. Click here for file

Additional file 3 **Scatterplots Human biopsies**. Same figure as Figures 1B-E, now showing extreme outliers. Click here for file

Additional file 4 **Mouse biopsies results**. Table showing the quality measure results for individual RNA samples isolated from mouse skin biopsies. Click here for file

Additional file 5 **Mouse biopsy sampling**. Overview of resulting holes on mouse skin after sampling with different punch diameters. Click here for file

Additional file 6 **Comparison to the gold standard methods**. Table showing the quality measure results of this study compared to the gold standard methods for RNA isolation from skin biopsies. Click here for file
