# Supplementary material for: RNA isolation for transcriptomics of human and mouse small skin biopsies
Source: BMC Res Notes. 2011 Oct 24;4:438. doi: 10.1186/1756-0500-4-438 (PMC3221605; doi:10.1186/1756-0500-4-438)
Supplement: Additional file 1 — RNA isolation protocol. Step-by-step protocol describing the isolation of RNA from skin biopsies. [file 1756-0500-4-438-S1.PDF]

Isolation of total RNA from skin biopsies

Materials
- Chloroform (ACS grade)
- Ethanol 70% (ACS grade)
- Liquid nitrogen
- Mortar and pestle
- Phase-Lock Gel Heavy (5 Prime – Cat. No. 2302870)
- Qiazol Lysis Reagent (Qiagen – Cat. No. 79306)
- RNeasy MinElute Cleanup kit (Qiagen – Cat. No. 74204)
- Heidolph REAX 2000 with multi-test tube attachment

Method
1. Place selected biopsy in a cryo-vial.
2. Quickly snap-freeze in liquid nitrogen.
3. Store @ -80℃.

Remark 1: Biopsies can be stored @ -80℃ for months or even years.

4. Place ~75-100 mg Phase-Lock Gel Heavy (PLG-H) in a 1.5 ml tube.

Remark 2: This can be done by filling a syringe with PLG-H and filling tubes using an analytical balance.

5. Pellet PLG-H @ 12,000xg for 30s.
6. Set tubes aside.

----

7. Grind biopsies to a fine powder in a pre-chilled mortar and pestle.
8. Add 300 µl Qiazol (Lysis Reagent) to a 1.5 ml tube.
9. Transfer powdered biopsy to this tube.
10. Vortex for 15s.
11. Shake tubes for 10 min on a Heidolph REAX 2000 with multi-test tube attachment (speed setting 6).
12. Spin down for 15s.
13. Add 60 µl Chloroform.
14. Vortex for 15s.
15. Keep @ RT for 3 min.
16. Spin down for 15s.
17. Transfer the (partially separated) mixture to a PLG-H containing tube.
18. Invert the tubes (do not vortex!).
19. Centrifuge @ 12,000xg for 15 min.
20. Transfer the aqueous phase to a new tube.

Remark 3: Next part of the protocol can also be found in the RNeasy MinElute Cleanup Handbook, "Appendix D: RNA Cleanup after Lysis and Homogenization with QIAzol® Lysis Reagent" on page 25.

21. Add 1 volume of 70% EtOH to the aqueous phase.
22. Vortex for 5s.
23. Transfer mixture to a RNeasy MinElute spin column.
24. Centrifuge @ 8,000xg for 15s.

25. Discard flow through.
26. Place the column in a new 2 ml collection tube.
27. Add 500 µl RPE buffer.
28. Centrifuge @ 8,000xg for 15s.
29. Discard flow through.
30. Add 500 µl 80% EtOH.
31. Centrifuge for @ 8,000xg 2 min.
32. Discard flow through.
33. Place column in a new 2 ml collection tube
34. Centrifuge @ full speed for 5 min.

Remark 4: The RNeasy MinElute manual states that the column lids should be open during centrifugation to evaporate residual ethanol. However, the lids tend to break at high speed. It is more convenient to close the lids and use the highest speeds your microcentrifuge can handle to remove the ethanol.

35. Place the column in a 1.5 ml collection tube.
36. Add 14 µl RNase-free water to the center of the column
37. Centrifuge @ full speed for 1 min to elute the RNA.

Remark 5: The end volume is ~12 µl.
Remark 6: The quantity of the RNA can be checked by a Nanodrop ND-1000 (Thermo Scientific) and the quality with the BioAnalyzer (Agilent Technologies – RNA 6000 Pico Chip kit).
