# Supplementary figures and images for: RNA isolation for transcriptomics of human and mouse small skin biopsies
Source: BMC Res Notes. 2011 Oct 24;4:438. doi: 10.1186/1756-0500-4-438 (PMC3221605; doi:10.1186/1756-0500-4-438)

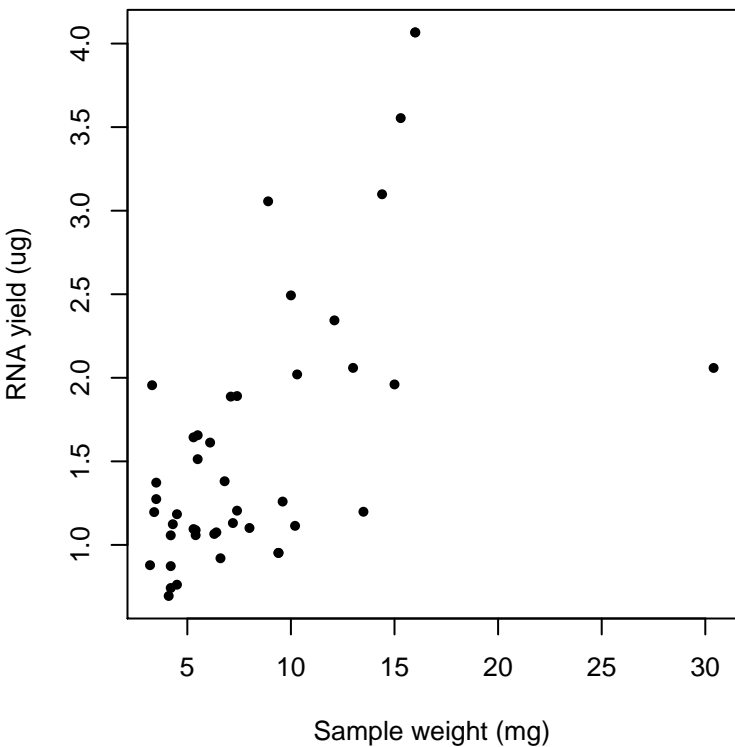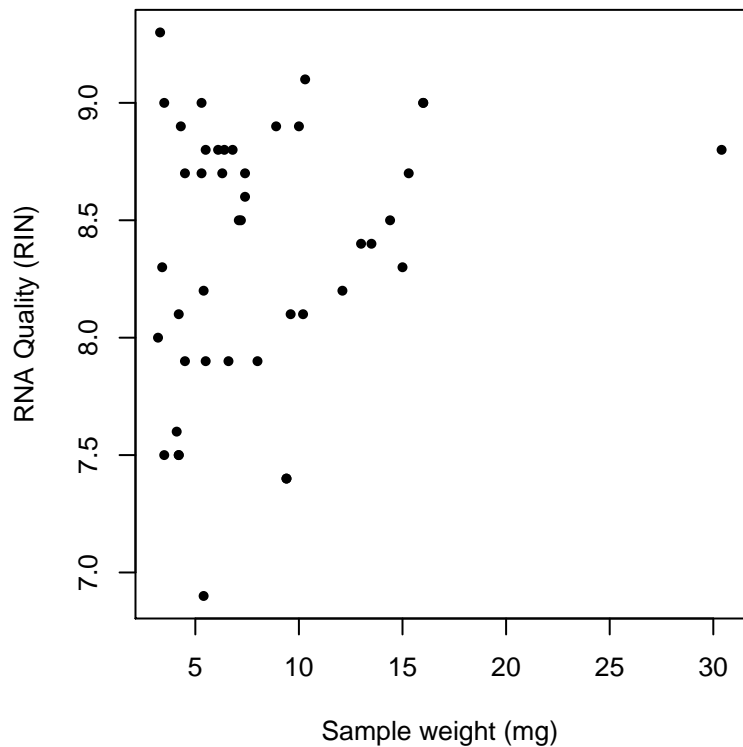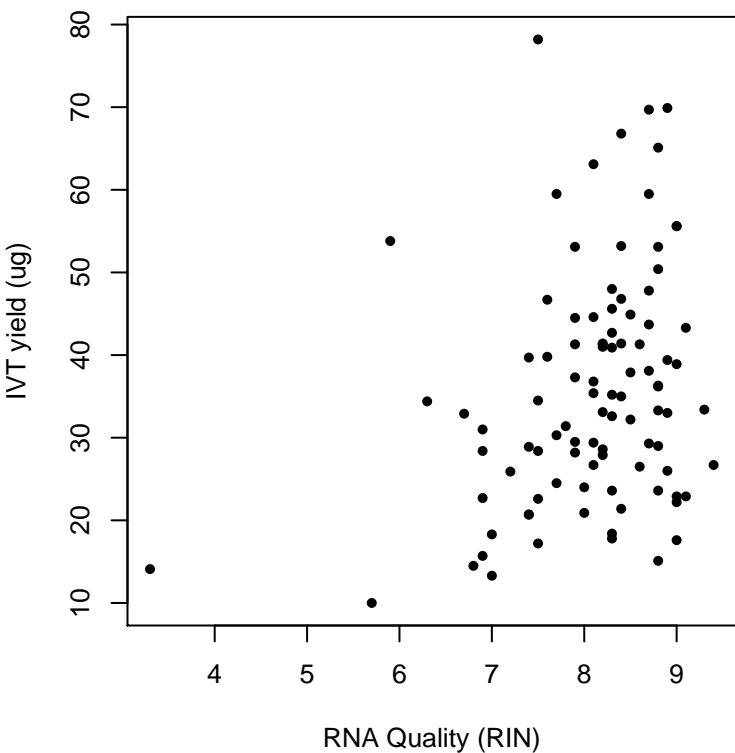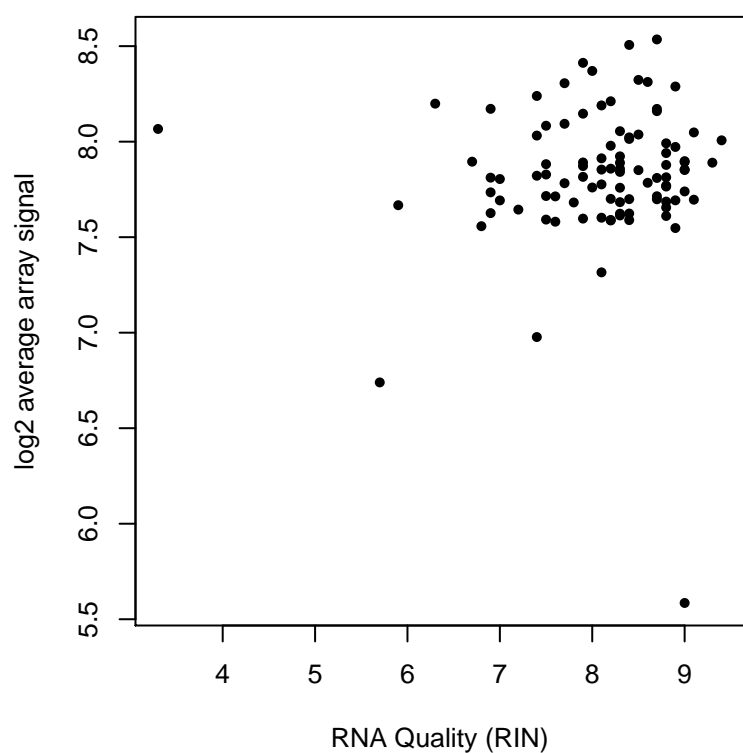

Supplement: Additional file 3 — Scatterplots Human biopsies. Same figure as Figures 1B-E, now showing extreme outliers. [file 1756-0500-4-438-S3.PDF]

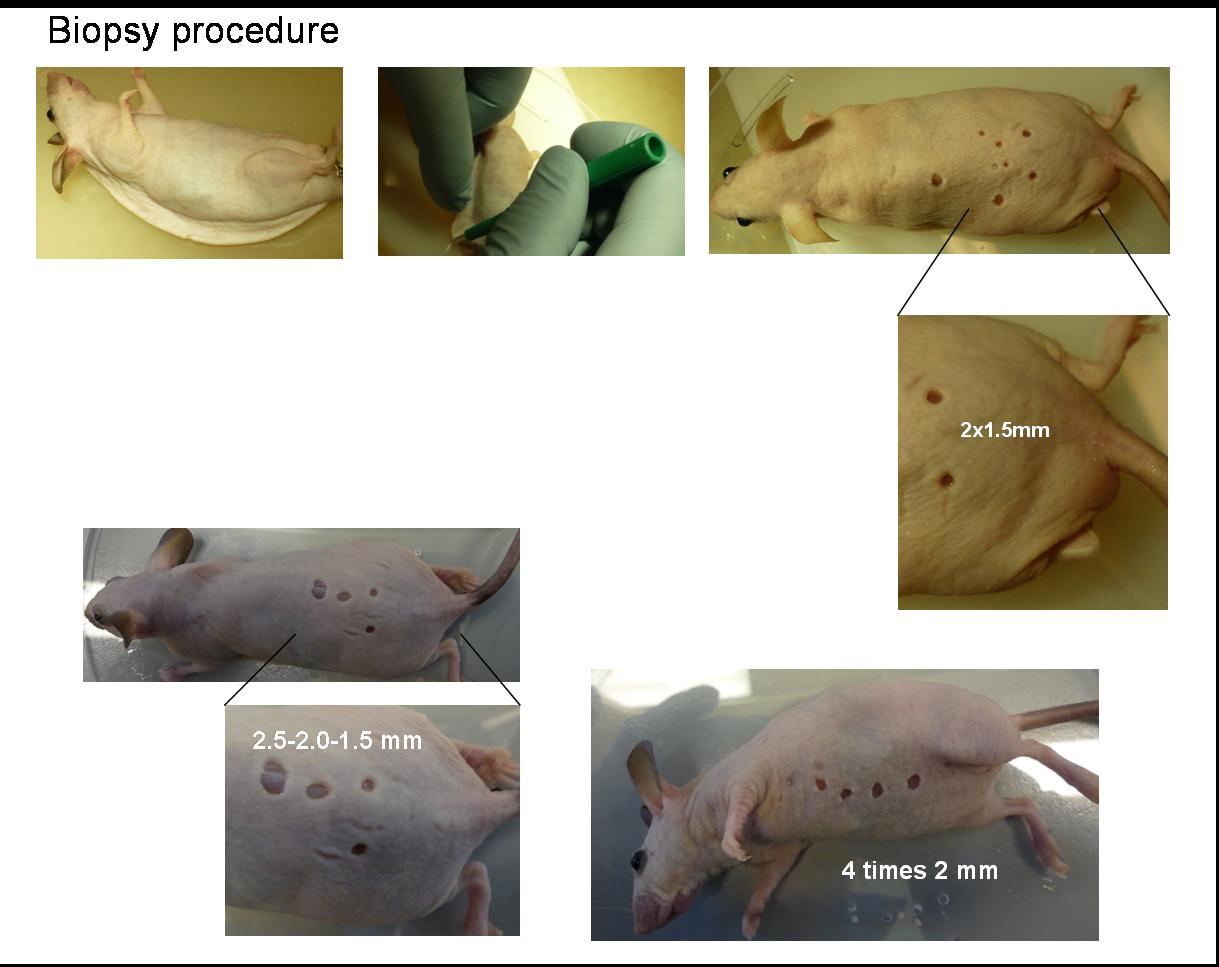

Supplement: Additional file 5 — Mouse biopsy sampling. Overview of resulting holes on mouse skin after sampling with different punch diameters. [file 1756-0500-4-438-S5.PNG]
